# Compensation of Horizontal Gravity Disturbances for High Precision Inertial Navigation

**DOI:** 10.3390/s18030906

**Published:** 2018-03-18

**Authors:** Junbo Tie, Juliang Cao, Meiping Wu, Junxiang Lian, Shaokun Cai, Lin Wang

**Affiliations:** National University of Defense Technology, Deya Road No. 109, Kaifu District, Changsha 410073, China; tiejunbo11@nudt.edu.cn (J.T.); meipingwu@263.net (M.W.); jx_lian@163.com (J.L.); csk527@163.com (S.C.); wanglinshanda@163.com (L.W.)

**Keywords:** inertial navigation, initial alignment, horizontal gravity disturbance, deflection of vertical

## Abstract

Horizontal gravity disturbances are an important factor that affects the accuracy of inertial navigation systems in long-duration ship navigation. In this paper, from the perspective of the coordinate system and vector calculation, the effects of horizontal gravity disturbance on the initial alignment and navigation calculation are simultaneously analyzed. Horizontal gravity disturbances cause the navigation coordinate frame built in initial alignment to not be consistent with the navigation coordinate frame in which the navigation calculation is implemented. The mismatching of coordinate frame violates the vector calculation law, which will have an adverse effect on the precision of the inertial navigation system. To address this issue, two compensation methods suitable for two different navigation coordinate frames are proposed, one of the methods implements the compensation in velocity calculation, and the other does the compensation in attitude calculation. Finally, simulations and ship navigation experiments confirm the effectiveness of the proposed methods.

## 1. Introduction

Inertial navigation systems (INSs) which measure the ship’s motion and constantly update the ship’s position are currently the main means of ship navigation. The performance of INS depends not only on the quality of inertial sensors, but also on the accuracy of the gravity information [1,2,3]. Even in the limiting situation of drift-free gyroscopes and perfect accelerometers, INS cannot be error-free, because uncertainties in the gravity model will produce errors. In recent years, significant improvements of INS, especially on the inertial sensors, have left horizontal gravity disturbances as the most important error sources of the navigation solution, particularly for rough topological areas [4,5]. In future highly accurate and long-endurance ship-mounted INS, cold atom interferometry gyroscopes will be used, with which the inertial-sensors-induced position error would be reduced to only a few meters per hour [5], so the compensation of horizontal gravity disturbances has to be considered.

Lots of previous works have been carried on the analysis of the errors induced by horizontal gravity disturbances. One of the earliest papers to discuss horizontal gravity disturbance-induced errors was by Levine and Gelb [6], who considered the horizontal gravity disturbance taken along the 35th parallel in the United States, representing the horizontal gravity disturbance by a first-order Gauss-Markov process, and evaluated the effect on INS with a steady-state solution of the error covariance matrix differential equation. Based on the covariance analysis method proposed by Levine and Gelb, several more sophisticated horizontal gravity disturbance models have been used to replace the first-order Gauss-Markov model to analyze the effect on INSs [7,8]. These analyses only focus on the effect of horizontal gravity disturbances on navigation calculation and ignore the effect on the initial alignment. The initial alignment and navigation calculation are two successive processes, and the effect of horizontal gravity disturbance are systematically and comprehensively analyzed by simultaneously considering the two successive processes in this paper.

Gradiometers can measure horizontal gravity disturbances in real-time and the measurement values can be used to compensate the INS, but the use of the needed precise instrument is very costly [9,10,11]. Nowadays, with the release of ultra-high degree Earth gravitational models, such as EGM2008, [12,13], horizontal gravity disturbances can be calculated through the Earth gravitational models with certain accuracy [14,15,16]. In this paper, the horizontal gravity disturbance used in simulation and ship experiment is calculated through EGM 2008 [12,13,14].

This paper is organized as follows: in Section 2, from the perspective of coordinate system and vector calculation, the effects of horizontal gravity disturbances on the initial alignment and navigation calculation are analyzed simultaneously. Two compensation methods suitable for two different navigation coordinate frames are proposed, and the method of compensation in velocity calculation is introduced in Section 3 and the method of compensation in attitude calculation is introduced in Section 4. The simulations and ship navigation experiment are discussed in Section 5, and the conclusions are provided in Section 6.

## 2. The Effect of DOV on Inertial Navigation System

### 2.1. Reference Coordinate Frames

Coordinate definition is the basis of inertial navigation. The first three coordinate systems are common ones and can be found in [17]. The last coordinate system is specially defined for the analysis in this paper:

*Earth-Centered-Earth-Fixed Frame* e: the origin of this coordinate frame is at the center of the Earth, whose z-axis points in the direction of the North pole, x-axis points towards the Greenwich Meridian, and y-axis completes the right-handed orthogonal frame. This frame rotates with the Earth with the rate $\omega_{ie}^{e}={\left[\begin{array}{ccc} 0 & 0 & \Omega \end{array}\right]}^{T}$, where Ω is the Earth’s rotation angular velocity.

*Body coordinate frame with Forward-Right-Down definition* b: this frame is defined based on the input axes of the inertial sensors and mounted on the ship. Its axes respectively point towards Forward-Right-Down of the ship.

**Figure 1:**
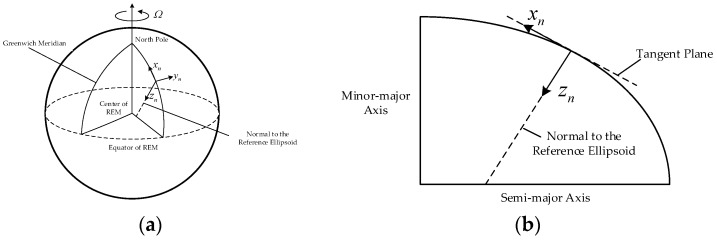
The definition of the navigation coordinate frame with North-East-Down definition. (**a**) is the definition of navigation coordinate frame with North-East-Down definition, (**b**) is the side view of (**a**).

*Navigation coordinate frame with North-East-Down definition* n: this frame is a local geodetic north-oriented, local-level coordinate frame as shown in Figure 1, the origin of this frame is at the position of the ship, and whose axes are denoted by $\left\{x_{n},y_{n},z_{n}\right\}$ and respectively point towards North, East, and Downward. It should be noted that $z_{n}$ is collinear with the normal to the reference ellipsoid.

**Figure 2:**
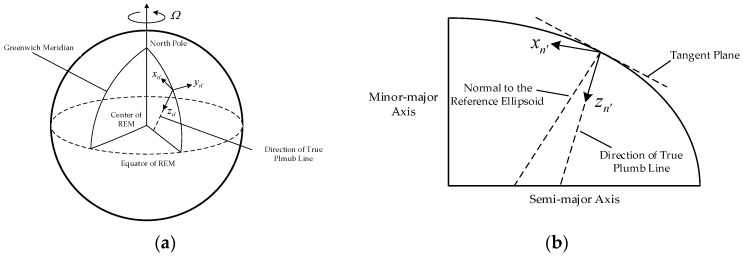
The definition of the navigation coordinate frame with true plumb line. (**a**) is the definition of navigation coordinate frame with true plumb line, (**b**) is the side view of (**a**).

*Navigation coordinate frame with true plumb line* $n^{\prime }$: this frame is similar to the n coordinate frame and is defined based on the direction of the real plumb line, whose axes are denoted by $\left\{x_{n^{\prime }},y_{n^{\prime }},z_{n^{\prime }}\right\}$ and $x_{n^{\prime }}$ also points towards north, but $z_{n^{\prime }}$ is collinear with the true plumb line and $y_{n^{\prime }}$ can be determined based on the right-hand rule, as shown in Figure 2.

### 2.2. Definition of Horizontal Gravity Disturbance

**Figure 3:**
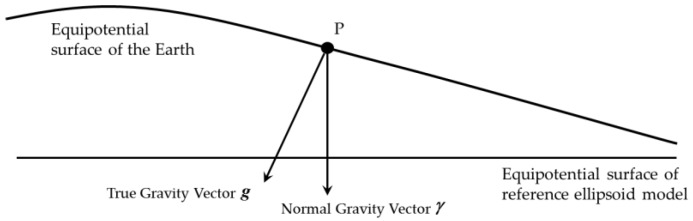
The definition of gravity disturbance vector, gravity disturbance and deflection of vertical.

According to the potential theory [3], the gravity vector is the perpendicular line of the equipotential surface of gravity. The Earth’s equipotential surface of gravity is very complex, and the equipotential surface of reference ellipsoid model, is used to approximate the Earth’s equipotential surface. As shown in Figure 3 [3], g is the true gravity vector of point P and γ is the normal gravity vector of point P, the gravity disturbance vector is the difference between the true gravity vector and the normal gravity vector. The difference in magnitude is the gravity disturbance and the difference in direction is the deflection of vertical (DOV). Due to DOV, there are some projected components of the true gravity vector in the horizontal plan, which are named horizontal gravity disturbance.

**Figure 4:**
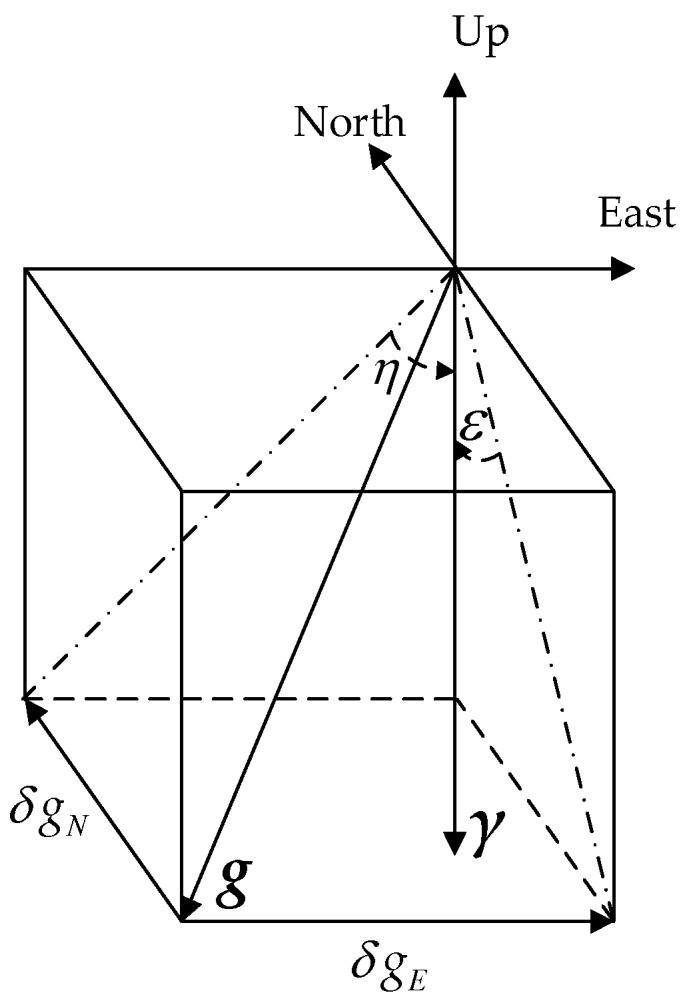
The definition of DOV.

DOV has two components, as shown in Figure 4 [3], a north component ξ and an east component η, the order of DOV component can maximally reach 100 arc seconds which is significantly larger than the modern accelerometer bias, whose typical value is 10 mGal (1 mGal = 10^−5^ m/s^2^), corresponding to a 2 arc seconds DOV [4].

Equation (1) shows the connections between DOV and horizontal gravity disturbance where γ is the norm of the normal gravity vector γ:

(1) $$\begin{array}{cc} \xi \approx -\frac{\Delta g_{N}}{\gamma } & \eta \approx -\frac{\Delta g_{E}}{\gamma } \end{array}$$

The normal gravity vector is in the direction of the normal to the reference ellipsoid [18], the connection among true gravity vector, normal gravity vector and horizontal gravity disturbance can be expressed in n frame as Equations (2)–(4):

(2) $$g^{n}=\gamma^{n}+\Delta g^{n}$$

(3) $$\gamma^{n}={\left[\begin{array}{ccc} 0 & 0 & \gamma \end{array}\right]}^{T}$$

(4) $$\Delta g^{n}={\left[\begin{array}{ccc} \Delta g_{N} & \Delta g_{E} & 0 \end{array}\right]}^{T}$$

where $g^{n}$ is the true gravity vector, and $\gamma^{n}$ is the normal gravity vector, and $\Delta g^{n}$ is the horizontal gravity disturbance. The superscript n means that these vectors are projected in n coordinate frame.

The horizonal gravity disturbance can be calculated through the spherical harmonic model (SHM) of the Earth’s gravity field as follows [3]:

(5) $$\Delta g_{N}=-\frac{GM}{r^{2}}\sum_{n=2}^{nmax}\sum_{m=0}^{n}{\left(\frac{a}{r}\right)}^{n}\left({\bar{C}}_{nm}^{*}\cdot \cos m\lambda +{\bar{S}}_{nm}\cdot \sin m\lambda \right)\frac{d{\bar{P}}_{nm}\left(\cos\vartheta \right)}{d\vartheta }$$

(6) $$\Delta g_{E}=\frac{GM}{\sin\vartheta \cdot r^{2}}\sum_{n=2}^{nmax}\sum_{m=0}^{n}{\left(\frac{a}{r}\right)}^{n}m\left[{\bar{C}}_{nm}^{*}\left(-\sin m\lambda \right)+{\bar{S}}_{nm}\left(\cos m\lambda \right)\right]{\bar{P}}_{nm}\left(\cos\vartheta \right)$$

where G is gravitational constant, M is the mass of the Earth, a is the major semi axis length of the reference ellipsoid, r is the radial distance from the calculated point to the center of the reference ellipsoid, ϑ is the geocentric colatitude of the calculated point, λ is the longitude of the calculated point, n and m are called the degree and order of the SHM, ${\bar{C}}_{nm}^{*}$ and ${\bar{S}}_{nm}$ are the coefficients of the SHM, *nmax* is the highest degree used in the SHM calculation and ${\bar{P}}_{nm}\left(\cos\vartheta \right)$ is the fully normalized Legendre functions of degree n and order m.

The fully normalized Legendre function is the core of SHM calculation and can be iteratively calculated as follows:

(7) $$\begin{array}{cc} {\bar{P}}_{nn}\left(\cos\vartheta \right)=\sqrt{\frac{2n+1}{2n}}\sin\vartheta {\bar{P}}_{n-1,n-1}\left(\cos\vartheta \right), & m=n,n\geq 2 \end{array}$$

(8) $$\begin{array}{cc} {\bar{P}}_{n,n-1}\left(\cos\vartheta \right)=\sqrt{2n+1}\cos\vartheta {\bar{P}}_{n-1,n-1}\left(\cos\vartheta \right), & m=n-1,n\geq 1 \end{array}$$

(9) $$\begin{array}{cc} {\bar{P}}_{nm}\left(\cos\vartheta \right)=\alpha_{nm}\cos\vartheta {\bar{P}}_{n-1,m}\left(\cos\vartheta \right)-\beta_{nm}{\bar{P}}_{n-2,m}\left(\cos\vartheta \right) & 0\leq m\leq n-2,n\geq 2 \end{array}$$

The coefficients and iteration initial values are given here:

(10) $$c_{nm}=\sqrt{\left(2n+1\right)/\left[\left(n-m\right)\left(n+m\right)\right]}$$

(11) $$\alpha_{nm}=\sqrt{\left(2n-1\right)}c_{nm}$$

(12) $$\beta_{nm}=\sqrt{\left(n+m-1\right)\left(n-m-1\right)/\left(2n-3\right)}c_{nm}$$

(13) $$\begin{array}{cc} {\bar{P}}_{0,0}\left(\cos\vartheta \right)=1 & {\bar{P}}_{1,1}\left(\cos\vartheta \right)=\sqrt{3}\sin\vartheta \end{array}$$

The iterative calculation formulas of the first-order derivative of the fully normalized Legendre function are given as below:

(14) $$\frac{d{\bar{P}}_{nn}\left(\cos\theta \right)}{d\theta }=n\cdot \cot\theta \cdot {\bar{P}}_{nn}\left(\cos\theta \right)$$

(15) $$\frac{d{\bar{P}}_{nm}\left(\cos\theta \right)}{d\theta }=n\cdot \cot\theta \cdot {\bar{P}}_{nm}\left(\cos\theta \right)-\;\frac{1}{\sin\theta }\frac{2n+1}{a_{nm}}{\bar{P}}_{n-1,m}\left(\cos\theta \right)$$

The iterative algorithm of the fully normalized Legendre function and its first-order derivative are listed in Table 1.

The SHM adopted in this paper is EGM2008 which is publicly released by the U.S. National Geospatial Intelligence Agency (NGA) EGM Development Team. This gravitational model is complete to spherical harmonic degree and order 2159 and contains additional coefficients extension to degree 2190 and order 2519. The full access to this model’s coefficients is provided in [19].

The true gravity vector will have a more concise expression in $n^{\prime }$ coordinate frame, because the $z_{n^{\prime }}$ is collinear with the true gravity vector. It’s the difference in direction rather than norm that affecting the accuracy of INS, so the approximation here is reasonable for the subject of this paper:

(16) $$g^{n^{\prime }}=\left[\begin{array}{c} 0\\ 0\\ \sqrt{\gamma^{2}+{\left(\Delta g_{N}\right)}^{2}+{\left(\Delta g_{E}\right)}^{2}} \end{array}\right]\approx \left[\begin{array}{c} 0\\ 0\\ \gamma \end{array}\right]$$

The concept of horizontal gravity disturbance compensation in this paper can be given here, that is horizontal gravity disturbance compensation means that the gravity vector used in INS calculation is the true gravity vector g rather than the normal gravity vector γ.

### 2.3. Formulas of INS

**Figure 5:**
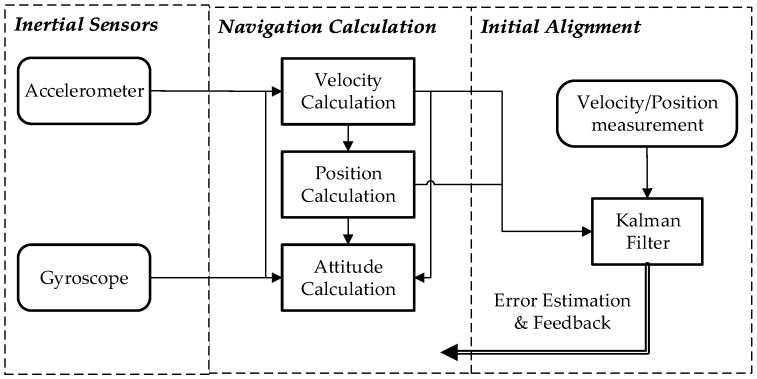
The schematic of inertial navigation.

Inertial navigation is an integration algorithm and can be decomposed into two successive steps as shown in Figure 5. The initial alignment is first executed to obtain the initial values of the integration algorithm, including initial attitudes, and initial velocities, and initial positions. Then the navigation calculation is executed based on the obtained initial values, including attitude calculation, velocity calculation, and position calculation. In fact, the execution of the first step contains the second step. Navigation calculation is first executed in initial alignment, then Kalman Filter (KF) recursion [20] is performed following the one-step navigation calculation [21]. After the KF measurement update, the estimated state can be feedback to fix the corresponding navigation calculation errors. Finally, the precise initial values will be obtained in the KF recursion. And the state equation and measurement equation of the KF used in initial alignment are given in Section 2.3.3.

#### 2.3.1. Attitude Calculation

Attitude information is used to project vectors into the target coordinate frame. The outputs of the inertial sensors are in b frame, while the navigation calculation is usually executed in n frame, so the outputs of the inertial sensors and other associated vectors should be transformed into n frame before they can be used. The direction cosine matrix (DCM) can be used to implement the coordinate transformation. The DCM between the n frame and b frame is a function of Euler angles [17]:

(17) $$C_{b}^{n}=\left[\begin{array}{ccc} \cos\theta \cos\psi & -\cos\phi \sin\psi +\sin\phi \sin\theta \cos\psi & \sin\phi \sin\psi +\cos\phi \sin\theta \cos\psi \\ \cos\theta \sin\psi & \cos\phi \cos\psi +\sin\phi \sin\theta \sin\psi & -\sin\phi \cos\psi +\cos\phi \sin\theta \sin\psi \\ -\sin\theta & \sin\phi \cos\theta & \cos\phi \cos\theta \end{array}\right]$$

where ϕ is the roll angle, θ is the pitch angle and ψ is the yaw angle.

The attitude calculation with DCM parameterization is given by [17]:

(18) $${\dot{C}}_{b}^{n}=C_{b}^{n}\left[\omega_{nb}^{b}\times \right]$$

where $\omega_{nb}^{b}$ is the body angular rate with respect to the navigation frame n and is given by [17]

(19) $$\omega_{nb}^{b}=\omega_{ib}^{b}-C_{n}^{b}\left(\omega_{ie}^{n}+\omega_{en}^{n}\right)$$

$\omega_{ib}^{b}$ is the body angular rate with respect to inertial frame and is measured by the gyroscopes. $\omega_{ie}^{n}$ is the earth rate projected in n frame and is given by [17]:

(20) $$\omega_{ie}^{n}={\left[\begin{array}{ccc} \Omega \cos L & 0 & -\Omega sinL \end{array}\right]}^{T}$$

L is the latitude of the INS, $\omega_{en}^{n}$ is the angular rate of navigation frame with respect to the earth frame projected in n frame, which is caused by the linear motion of the INS on the ellipsoidal surface. The formulation of $\omega_{en}^{n}$ in n frame is given by [17]:

(21) $$\omega_{en}^{n}={\left[\begin{array}{ccc} \frac{v_{E}}{R_{N}+h} & \frac{-v_{N}}{R_{M}+h} & \frac{-v_{E}\tan L}{R_{N}+h} \end{array}\right]}^{T}$$

$R_{M}$ and $R_{N}$ are the meridian and transverse radius of the ellipsoid curvature, respectively. $v_{E}$ and $v_{N}$ are east and north component of the velocity, respectively. h is the height of the ship relative to the reference ellipsoid.

#### 2.3.2. Velocity Calculation and Position Calculation

The velocity calculation equation is usually derived under n coordinate framework [17]:

(22) $${\dot{v}}_{e}^{n}=C_{b}^{n}f^{b}-\left(2\omega_{ie}^{n}+\omega_{en}^{n}\right)\times v_{e}^{n}+g^{n}$$

where $v_{e}^{n}$ is the velocity of INS relative to the Earth, $C_{b}^{n}$ transforms the measured specific force $f^{b}$ measured by accelerometers into navigation coordinate n from body coordinate b. It should be noted that if the INS is without compensation, the normal gravity vector is used in the velocity calculation. In practice the velocity calculation equation is Equation (23):

(23) $${\dot{v}}_{e}^{n}=C_{b}^{n}f^{b}-\left(2\omega_{ie}^{n}+\omega_{en}^{n}\right)\times v_{e}^{n}+\gamma^{n}$$

The position calculation is given here [17] and the calculation of height is not considered here, because the vertical channel of INS is divergent [18], and λ denotes the longitude of INS:

(24) $$\dot{L}=\frac{1}{R_{M}+h}v_{N}$$

(25) $$\dot{\lambda }=\frac{\sec L}{R_{N}+h}v_{E}$$

#### 2.3.3. Initial Alignment

In general, the initial alignment is executed when the ship is docked at the wharf or mooring, so the true velocity of the INS is approximately equal to zero. Then the non-zero velocity output of INS is the velocity error of INS, and can be used as the measurement of KF to estimate the corresponding errors of INS. The KF in initial alignment is built based on the error equations of INS. The attitude error equation is given by [17]:

(26) $$\Psi ={\left[\begin{array}{ccc} \delta \phi & \delta \theta & \delta \psi \end{array}\right]}^{T}$$

(27) $$\dot{\Psi }=-\left(\omega_{ie}^{n}+\omega_{en}^{n}\right)\times \Psi +\delta \omega_{en}^{n}-C_{b}^{n}\delta \omega_{ib}^{b}$$

where Ψ is the attitude error vector, δϕ is the roll error, δθ is the pitch error, δψ is the yaw error, $\delta \omega_{en}^{n}$ is turn rate error and $\delta \omega_{ib}^{b}$ is the measurement noise of gyroscopes.

The velocity error equation is given in [17]:

(28) $$\delta v_{e}^{n}=\left[\begin{array}{ccc} \delta v_{N} & \delta v_{E} & \delta v_{D} \end{array}\right]$$

(29) $$\delta {\dot{v}}_{e}^{n}=\left[f^{n}\times \right]\Psi -\left(2\omega_{ie}^{n}+\omega_{en}^{n}\right)\times \delta v_{e}^{n}-\delta \omega_{en}^{n}\times v_{e}^{n}+C_{b}^{n}\delta f^{b}$$

where $\delta v_{e}^{n}$ is the velocity error vector, including three components, $\delta v_{N}$ is the north velocity error, $\delta v_{E}$ is the east velocity error, $\delta v_{D}$ is the downward velocity error, $\delta f^{b}$ is the measurement noise of accelerometers, and $\left[f^{n}\times \right]$ is a skew matrix of the specific force vector:

(30) $$\left[f^{n}\times \right]=\left[\begin{array}{ccc} 0 & -f_{D} & f_{E}\\ f_{D} & 0 & -f_{N}\\ -f_{E} & f_{N} & 0 \end{array}\right]$$

where $f_{N}$ is the north component of the specific force, $f_{E}$ is the east component of the specific force, and $f_{D}$ is the downward component of the specific force.

Because of the instability of INS’s vertical channel, five states are generally used to describe the error propagation of INS, including two horizontal velocity errors, and attitude errors. The state equation of KF is given in [22]:

(31) $$x={\left[\begin{array}{ccccc} \delta \phi & \delta \theta & \delta \psi & \delta v_{N} & \delta v_{E} \end{array}\right]}^{T}$$

(32) $$\dot{x}=Fx+q=\left[\begin{array}{cc} F_{aa} & F_{av}\\ F_{va} & F_{vv} \end{array}\right]x+q$$

where F is the system equation, q is the model noise vector, including measurement noise of accelerometers and measurement noise of gyroscopes.

(33) $$F_{aa}=\left[\begin{array}{ccc} 0 & -\left(\begin{array}{l} \Omega \sin L+\\ \frac{v_{E}}{R_{N}}\tan L \end{array}\right) & \frac{v_{N}}{R_{M}}\\ \left(\begin{array}{l} \Omega \sin L+\\ \frac{v_{E}}{R_{N}}\tan L \end{array}\right) & 0 & \left(\begin{array}{l} \Omega \cos L+\\ \frac{v_{E}}{R_{N}} \end{array}\right)\\ \frac{-v_{N}}{R_{M}} & -\left(\begin{array}{l} \Omega \cos L\\ +\frac{v_{E}}{R_{N}} \end{array}\right) & 0 \end{array}\right]$$

(34) $$F_{av}=\left[\begin{array}{cc} 0 & \frac{1}{R_{N}}\\ -\frac{1}{R_{M}} & 0\\ 0 & -\frac{\tan L}{R_{N}} \end{array}\right]$$

(35) $$F_{va}=\left[\begin{array}{ccc} 0 & -f_{D} & f_{E}\\ f_{D} & 0 & -f_{N} \end{array}\right]$$

(36) $$F_{vv}=\left[\begin{array}{cc} \frac{v_{D}}{R_{M}} & -2\left(\begin{array}{l} \Omega \sin L+\\ \frac{v_{E}}{R_{N}}\tan L \end{array}\right)\\ \left(\begin{array}{l} 2\Omega \sin L+\\ \frac{v_{E}}{R_{N}}\tan L \end{array}\right) & \frac{1}{R_{N}}\left(v_{N}\tan L+v_{D}\right) \end{array}\right]$$

To update the state vector estimation with a set of measurements, it is necessary to know how the measurements vary with the states. This is the function of the measurement model. The velocity errors of INS are chosen as the measurements, and the measurement equation is Equation (37):

(37) $$\left[\begin{array}{c} \delta v_{N}\\ \delta v_{E} \end{array}\right]=Hx=\left[\begin{array}{ccccc} 0 & 0 & 0 & 1 & 0\\ 0 & 0 & 0 & 0 & 1 \end{array}\right]x+v$$

When the KF recursion is completed, the initial states of INS, especially the initial attitudes, are obtained. The navigation coordinate frame n is the frame in which navigation calculation is executed. Although the n frame is theoretically determined as defined in Section 2.1, the n frame of the initial position is unknown before the initial alignment. While the b frame is known, because the outputs of inertial sensors are in the b frame. In order to determine the n frame, the connection between the two frames, namely $C_{b}^{n}$, is calculated in the initial alignment, then the n frame can be built.

### 2.4. Effect of Horizontal Gravity Disturbance on INS

The effect of horizontal gravity disturbance on INS will be analyzed from the perspective of the coordinate system and vector calculation:

*Law of vector calculation*: all vectors associated in the calculation must be projected into the same coordinate frame. According to the law, there will be two constraints on the calculation of INS:

(I) The navigation coordinate frame built in the initial alignment must be consistent with the navigation coordinate frame in which the navigation calculation is implemented.
(II) The vectors used in navigation calculation must be projected into the same navigation coordinate frame.

The navigation coordinate frame built in the initial alignment must be consistent with the navigation coordinate frame in which the navigation calculation is implemented.

The vectors used in navigation calculation must be projected into the same navigation coordinate frame.

When the velocity calculation is executed without compensation of horizontal gravity disturbance, the gravity vector used in velocity calculation is the normal gravity vector as Equation (3) rather than the true gravity vector as Equation (2), then there will be a systematic error in the velocity calculation due to the inaccurate gravity information. This is the traditional explanation for the effect of horizontal gravity disturbance on INS, and can be found in [6,14,23,24,25].

Another explanation is given here from the perspective of coordinate system and vector calculation, and the compensation methods proposed in this paper are just derived based on this explanation. Comparing Equation (3) with Equation (16), it can be found that the true gravity vector in $n^{\prime }$ frame has the same form as the normal gravity vector in n frame. When the velocity calculation is executed without compensation, using the normal gravity in n frame as Equation (3) can be regarded as using the true gravity vector in $n^{\prime }$ frame as Equation (16) in the velocity calculation, and this calculation doesn’t meet constraint (II), then velocity error will arise. In addition, due to the violation of constraint (II), the velocity error Equation (29) is unable to accurately describe the velocity error dynamics, and there should be an extra gravity-induced term in the velocity error equation. That’s to say, there exists a model error in the state equation of KF and the correct velocity error equation should be Equation (38).

(38) $$\delta {\dot{v}}_{e}^{n}=\left[f^{n}\times \right]\Psi -\left(2\omega_{ie}^{n}+\omega_{en}^{n}\right)\times \delta v_{e}^{n}-\delta \omega_{en}^{n}\times v_{e}^{n}+C_{b}^{n}\delta f^{b}+\Delta g^{n}$$

Subtracting Equation (29) from Equation (38), the estimation error which is due to the model error can be obtained.

(39) $$\Delta {\dot{v}}_{e}^{n}=\left[f^{n}\times \right]\Delta \Psi +\Delta g^{n}$$

The Kalman filter will converge when the observation reaches zero [20], and the observation here is the velocity error of INS, and the attitude estimation error due to the horizontal gravity disturbance can be obtained by setting Equation (39) equal to zero.

(40) $$\Delta \Psi ={\left[\begin{array}{ccc} -\eta & \xi & 0 \end{array}\right]}^{T}$$

Due to ΔΨ the attitude error estimation obtained in the initial alignment diverges from its true value. The navigation coordinate built in the initial alignment is not consistent with the navigation coordinate frame in which the navigation calculation is executed, and this is the violation of constraint (I).

In our opinion, this is due to the violations of constraints on the calculation of INS that causes the adverse effect of the horizontal gravity disturbance on INS. From this point of view, ensuring the constraints being satisfied is the crucial issue of the horizontal gravity disturbance compensation.

When n frame being the navigation coordinate frame, the normal gravity vector should be converted to the true gravity vector with DOV information, and the true gravity vector should be used in velocity calculation to satisfy the constraints. This method is called compensation in velocity calculation and is provided in Section 3.

In addition, $n^{\prime }$ can also be chosen as the navigation coordinate frame, and the attitude calculation is compensated with DOV information, and the benefit of using this navigation coordinate frame is the concise form of true gravity vector, as Equation (16). This method is called compensation in attitude calculation and provided in Section 4.

## 3. Compensation in Velocity Calculation

**Figure 6:**
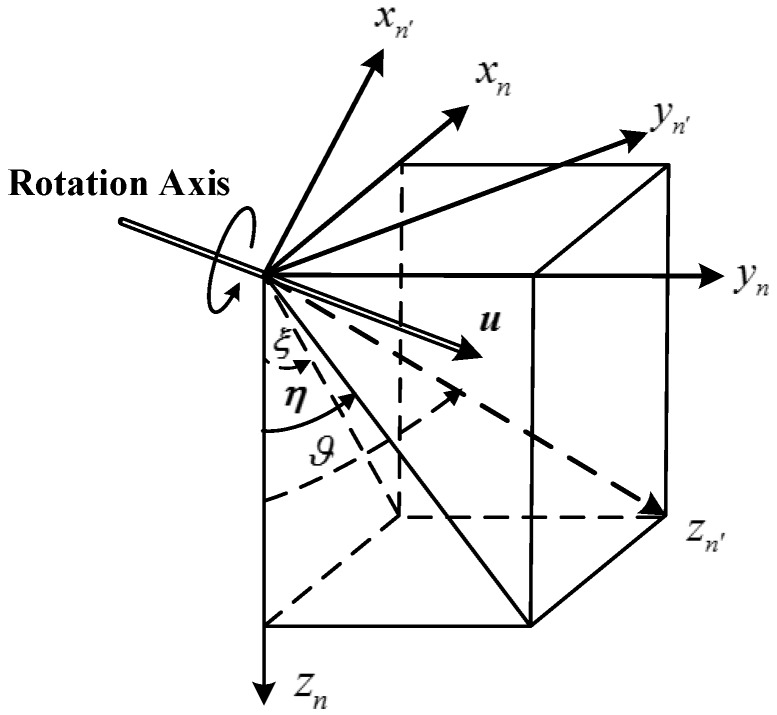
The geometric relation between the two navigation coordinate frames.

Compensation in velocity calculation is to convert the normal gravity vector to obtain the true gravity vector in the n coordinate frame with DOV information. The true gravity vector in the $n^{\prime }$ coordinate has a concise form as Equation (16), the true gravity vector in n frame can be obtained as follows:

(41) $$g^{n}=C_{n^{\prime }}^{n}g^{n^{\prime }}$$

where $C_{n^{\prime }}^{n}$ is a DCM which can transform vectors from $n^{\prime }$ to n, and this DCM can be calculated based on the geometrical relationship between the two navigation coordinate frames. As shown in Figure 6, $z_{n}$ is aligned with the normal gravity vector and $z_{n^{\prime }}$ is aligned with the true gravity vector, and the geometrical relation between the two navigation coordinate frames is determined by DOV. $n^{\prime }$ frame can be obtained through rotating the coordinate frame n by ϑ along the rotational axis u.

It is obvious that the rotational axis and rotational angle are associated with the DOV components and can be determined as follows. The rotation axis u satisfies four constraints:

(1) In the plane $x_{n}-y_{n}$;
(2) Pass through the origin of the two coordinate frames;
(3) Be orthogonal to the plane $z_{n}-z_{n^{\prime }}$;
  u **is the unit vector;**

Then rotational u axis can be determined based on the four constraints:

(42) $$u={\left[\begin{array}{ccc} \frac{-\eta }{\sqrt{\xi^{2}+\eta^{2}}} & \frac{\xi }{\sqrt{\xi^{2}+\eta^{2}}} & 0 \end{array}\right]}^{T}$$

The rotational angle ϑ is the included angle between $z_{n}$ and $z_{n^{\prime }}$, and the direction vectors of $z_{n}$ and $z_{n^{\prime }}$ are respectively ${\left[\begin{array}{ccc} 0 & 0 & 1 \end{array}\right]}^{T}$ and ${\left[\begin{array}{ccc} \xi & \eta & 1 \end{array}\right]}^{T}$ in n frame, then the included angle ϑ can be obtained based on the product of the two direction vectors:

(43) $$\vartheta =\frac{1}{\sqrt{1+\xi^{2}+\eta^{2}}}$$

The quaternion which describes the geometric relation between the two coordinate frames can be determined based on the rotational axis u and rotational angle ϑ:

(44) $$\begin{array}{l} Q={\left[\begin{array}{cccc} q_{0} & q_{1} & 0 & q_{2} \end{array}\right]}^{T}\\ q_{0}=\cos\left(\arccos\left(\frac{1}{\sqrt{1+\xi^{2}+\eta^{2}}}\right)/2\right)\\ q_{1}=\sin\left(\arccos\left(\frac{1}{\sqrt{1+\xi^{2}+\eta^{2}}}\right)/2\right)\cdot \left(\frac{-\eta }{\sqrt{\xi^{2}+\eta^{2}}}\right)\\ q_{2}=\sin\left(\arccos\left(\frac{1}{\sqrt{1+\xi^{2}+\eta^{2}}}\right)/2\right)\cdot \left(\frac{\xi }{\sqrt{\xi^{2}+\eta^{2}}}\right) \end{array}$$

According to the connection between quaternion and DCM [17], $C_{n^{\prime }}^{n}$ can be calculated as follows:

(45) $$C_{n^{\prime }}^{n}=\left[\begin{array}{ccc} q_{0}^{2}+q_{1}^{2}-q_{2}^{2} & -2q_{0}\cdot q_{2} & 2q_{1}\cdot q_{2}\\ 2q_{0}\cdot q_{2} & q_{0}^{2}-q_{1}^{2}-q_{2}^{2} & -2q_{0}\cdot q_{1}\\ 2q_{1}\cdot q_{2} & 2q_{0}\cdot q_{1} & q_{0}^{2}-q_{1}^{2}+q_{2}^{2} \end{array}\right]$$

Finally, the true gravity vector in n frame is obtained through Equations (41) and (45). Using this true gravity vector in velocity calculation to meet the two constraints on INS, then the adverse impact of horizontal gravity disturbance on INS will be eliminated.

## 4. Compensation in Attitude Calculation

As $n^{\prime }$ frame being the navigation coordinate frame, the vectors used in velocity calculation should be projected as Equation (46):

(46) $${\dot{v}}_{e}^{n^{\prime }}=C_{b}^{n^{\prime }}f^{b}-\left(2\omega_{ie}^{n^{\prime }}+\omega_{en^{\prime }}^{n^{\prime }}\right)\times v_{e}^{n^{\prime }}+g^{n^{\prime }}$$

The vectors in the above equation are the counterparts of the vectors in Equation (22), and the difference is that the vectors here are projected in $n^{\prime }$ frame rather than n frame. And the $n^{\prime }$ frame is determined through DCM $C_{b}^{n^{\prime }}$ which is updated with Equation (47).

(47) $${\dot{C}}_{b}^{n^{\prime }}=C_{b}^{n^{\prime }}\left[\omega_{n^{\prime }b}^{b}\times \right]$$

where $\left[\omega_{n^{\prime }b}^{b}\times \right]$ is the skew matrix of $\omega_{n^{\prime }b}^{b}$, and $\omega_{n^{\prime }b}^{b}$ is the turn rate of $n^{\prime }$ frame relative to b frame. Calculation of $\omega_{n^{\prime }b}^{b}$ can be decomposed into three parts:

(48) $$\omega_{n^{\prime }b}^{b}=\omega_{ib}^{b}-{\left(C_{b}^{n\prime }\right)}^{T}\cdot \left(\omega_{ie}^{n^{\prime }}+\omega_{en}^{n^{\prime }}\right)-\omega_{nn^{\prime }}^{n^{\prime }}$$

where $\omega_{ib}^{b}$ is the output of gyroscopes, $\omega_{ie}^{n^{\prime }}$ and $\omega_{en}^{n^{\prime }}$ can be calculated based on the position and velocity provided by INS as follows:

(49) $$\omega_{ie}^{n^{\prime }}={\left(C_{n^{\prime }}^{n}\right)}^{T}\cdot \omega_{ie}^{n}$$

(50) $$\omega_{ie}^{n}={\left[\begin{array}{ccc} \Omega \cdot \cos L & 0 & -\Omega \cdot \sin L \end{array}\right]}^{T}$$

(51) $$\omega_{en}^{n^{\prime }}={\left(C_{n^{\prime }}^{n}\right)}^{T}\cdot \omega_{en}^{n}$$

(52) $$\omega_{en}^{n}={\left[\begin{array}{ccc} \frac{v_{E}}{R_{N}+h} & \frac{-v_{N}}{R_{M}+h} & \frac{-v_{E}\cdot \tan L}{R_{N}+h} \end{array}\right]}^{T}$$

$\omega_{nn^{\prime }}^{n^{\prime }}$ is the turn rate of $n^{\prime }$ coordinate frame relative to n coordinate frame. DOV will change as INS moving on the Earth’s surface, and $\omega_{nn^{\prime }}^{n^{\prime }}$ just indicates the changes of DOV. $\omega_{nn^{\prime }}^{n^{\prime }}$ can be obtained based on the quaternion multiplication as Equation (53).

The quaternion can be updated with Equations (53)–(56), and ⊗ denotes the quaternion multiplication operator [17]:

(53) $$Q\left(t_{k}\right)=Q\left(t_{k-1}\right)⊗q\left(T\right)$$

(54) $$\begin{array}{cc} Q\left(t_{k}\right)={\left[\begin{array}{cccc} q_{0} & q_{1} & q_{2} & q_{3} \end{array}\right]}^{T} & Q\left(t_{k-1}\right)={\left[\begin{array}{cccc} {q^{\prime }}_{0} & {q^{\prime }}_{1} & {q^{\prime }}_{2} & {q^{\prime }}_{3} \end{array}\right]}^{T} \end{array}$$

(55) $$Q\left(t_{k}\right)=M\left[Q\left(t_{k-1}\right)\right]\cdot q\left(T\right)$$

(56) $$M\left[Q\left(t_{k-1}\right)\right]=\left[\begin{array}{cccc} {q^{\prime }}_{0} & -{q^{\prime }}_{1} & -{q^{\prime }}_{2} & -{q^{\prime }}_{3}\\ {q^{\prime }}_{1} & {q^{\prime }}_{0} & -{q^{\prime }}_{3} & {q^{\prime }}_{2}\\ {q^{\prime }}_{2} & {q^{\prime }}_{3} & {q^{\prime }}_{0} & -{q^{\prime }}_{1}\\ {q^{\prime }}_{3} & -{q^{\prime }}_{2} & {q^{\prime }}_{1} & {q^{\prime }}_{0} \end{array}\right]$$

where q(T) denotes the quaternion change caused by $\omega_{nn^{\prime }}^{n^{\prime }}$, and $T=t_{k}-t_{k-1}$ is the update interval. Because DOV changes very slowly compared with the update interval of INS, $\omega_{nn^{\prime }}^{n^{\prime }}$ can be assumed to remain constant during the update interval, the integral of $\omega_{nn^{\prime }}^{n^{\prime }}$ which is the rotational vector can be obtained as follows:

(57) $$\Theta =\int_{t_{k-1}}^{t_{k}}\omega_{nn^{\prime }}^{n^{\prime }}\left(t\right)dt=T\cdot \omega_{nn^{\prime }}^{n^{\prime }}\left(t_{k-1}\right)$$

(58) $$\Theta ={\left[\begin{array}{ccc} \Theta_{x} & \Theta_{y} & \Theta_{z} \end{array}\right]}^{T}$$

q(T) can be constructed from the rotational vector [17]:

(59) $$q\left(T\right)=\cos\frac{\left\|\Theta \right\|}{2}+\frac{\Theta }{\left\|\Theta \right\|}\sin\frac{\left\|\Theta \right\|}{2}=\left[\begin{array}{c} \cos\left(\left\|\Theta \right\|/2\right)\\ \Theta_{x}\sin\left(\left\|\Theta \right\|/2\right)\\ \Theta_{y}\sin\left(\left\|\Theta \right\|/2\right)\\ \Theta_{z}\sin\left(\left\|\Theta \right\|/2\right) \end{array}\right]$$

$Q\left(t_{k}\right)$ and $Q\left(t_{k-1}\right)$ are calculated by substituting DOV into Equation (44), then $Q\left(t_{k}\right)$ and $Q\left(t_{k-1}\right)$ are substituted into Equation (44) for solving q(T), then $\omega_{nn^{\prime }}^{n^{\prime }}$ can be obtained based on Equations (57)–(59):

(60) $$\omega_{nn^{\prime }}^{n^{\prime }}=\left[\begin{array}{c} \left(\left\|\Theta \right\|/T\right)\cdot \Theta_{x}\\ \left(\left\|\Theta \right\|/T\right)\cdot \Theta_{y}\\ \left(\left\|\Theta \right\|/T\right)\cdot \Theta_{z} \end{array}\right]$$

## 5. Simulation and Shipborne Inertial Navigation Experiment

### 5.1. Initial Alignment Simulation

In this subsection, simulations are used to verify the compensation effect of proposed methods in initial alignment. All inertial sensors exhibit noise from a number of sources [22] and the accuracy ranges of inertial sensors are set as Table 2, which is a concise classification of accuracy given in [26]. Horizontal gravity disturbance has more significant effect on high-precision INS, and gravity disturbance compensation has little improvement on the accuracy of low- and medium-precision INS for the errors of inertial sensors being much larger than the horizontal gravity disturbance. In this simulation, the inertial sensors are assumed to be the high precise devices.

The horizontal gravity disturbance at the initial position is set to the mean value of the horizontal gravity disturbance on the ship’s trajectory in the next section. The latitude, longitude and height of the ship is provided by GNSS and substituted into EGM2008 to calculate the horizontal gravity disturbance on the ship’s trajectory as described in Section 2.2. And the mean north component of the horizontal gravity disturbance is −17.94 mGal and the mean east component of the horizontal gravity disturbance is 34.66 mGal. The corresponding DOV can be calculated based on Equation (1).

**Figure 7:**
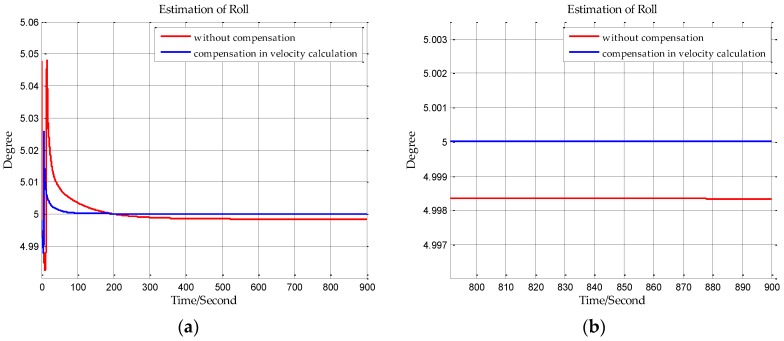
(**a**) is the estimation of roll with compensation in velocity calculation, and (**b**) is the enlarged view of (**a**) in the last 100 s.

**Figure 8:**
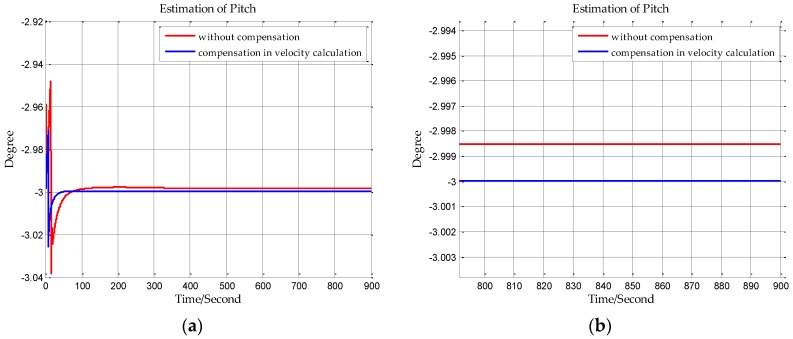
(**a**) is the estimation of pitch with compensation in velocity calculation, and (**b**) is the enlarged view of (**a**) in the last 100 s.

**Figure 9:**
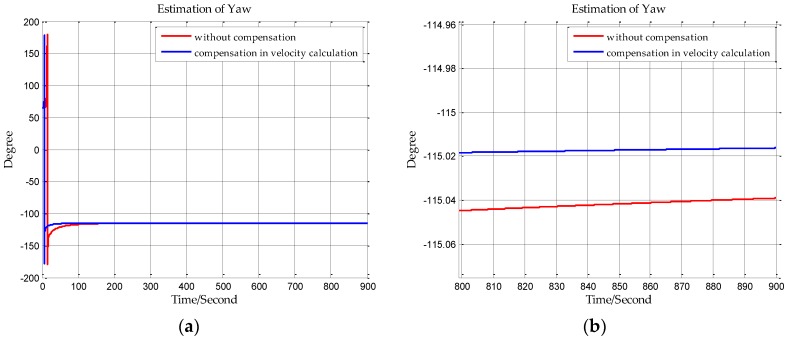
(**a**) is the estimation of yaw with compensation in velocity calculation, and (**b**) is the enlarged view of (**a**) in the last 100 s.

**Figure 10:**
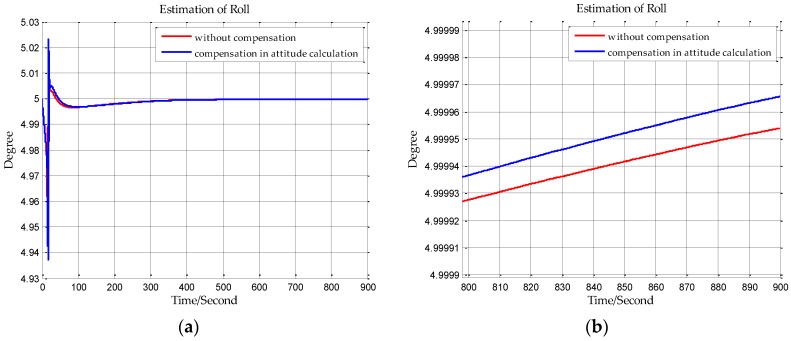
(**a**) is the estimation of roll with compensation in attitude calculation, and (**b**) is the enlarged view of (**a**) in the last 100 s.

**Figure 11:**
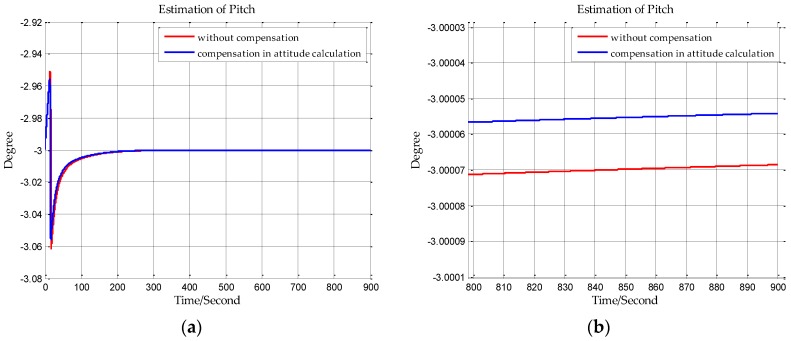
(**a**) is the estimation of pitch with compensation in attitude calculation, and (**b**) is the enlarged view of (**a**) in the last 100 s.

**Figure 12:**
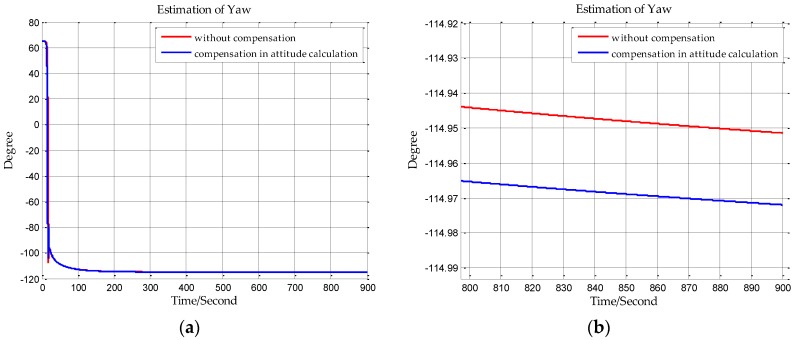
(**a**) is the estimation of yaw with compensation in attitude calculation, and (**b**) is the enlarged view of (**a**) in the last 100 s.

The sampling frequency of inertial sensors and the time of initial alignment are two important parameters in this simulation. The sampling frequency of the shipborne inertial navigation in the next section is 200 Hz, so we set the sampling frequency to be 200 Hz in this simulation. And the time of initial alignment is designed with the principle that the time of initial alignment must ensure that the Kalman filter fully converges. Once the Kalman filter has converged, continuing to extend the alignment time will not improve the estimation accuracy. Based on our practical experience, the time of initial alignment is set to 15 min which is enough for most inertial navigation systems. In addition, it can be seen from the following simulation results in Figure 7, Figure 8, Figure 9, Figure 10, Figure 11 and Figure 12 that the estimations of the Euler angles have fully converged within 10 min, so setting the time of initial alignment to be 15 min is appropriate here. The true values of the initial states of INS are listed in Table 3, and these parameters are set with reference to the initial states of INS in the ship experiment of the next section.

When n frame is the navigation coordinate frame, the estimation results of the Euler angles are shown from Figure 7, Figure 8 and Figure 9. When $n^{\prime }$ frame is the navigation coordinate frame, the estimation results of the Euler angles are shown is Figure 10, Figure 11 and Figure 12 and the estimation accuracy comparisons of the simulations are listed in Table 4 and Table 5. In Table 4, the estimation accuracy of roll is increased by 5.90 arc seconds, and the estimation accuracy of pitch is increased by 5.26 arc seconds, and the estimation accuracy of yaw is increased by 82.33 arc seconds. In Table 5, the estimation accuracy of roll is increased by 0.072 arc seconds, and the estimation accuracy of pitch is increased by 0.25 arc seconds, and the estimation accuracy of yaw is increased by 73.98 arc seconds. It can be concluded from the simulation results that the estimation accuracy of attitudes in initial alignment is more accurate with compensation.

### 5.2. Shipborne Inertial Navigation Experiment

**Figure 13:**
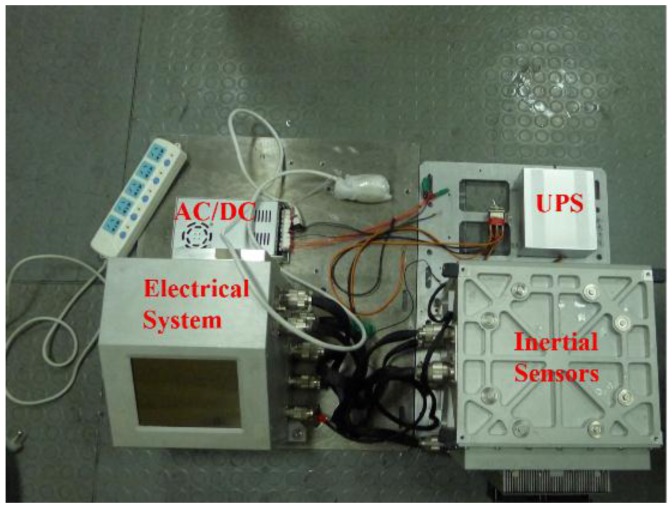
The ship mounted strapdown inertial navigation system in this experiment.

A ship navigation experiment is used to verify the effectiveness of the proposed methods, and the ship mounted instruments consist of a high-precision strapdown inertial navigation system (SINS) and a Novatel^®^ GNSS receiver contained in the electric system of SINS as shown in Figure 13. This SINS is developed by the author’s group, whose inertial sensors are high precision ring laser gyroscopes and quartz flexible accelerometers. Based on the long term static test, the bias stability of the ring laser gyroscope is about 0.004°/h and the bias repeatability of the accelerometer is about 0.59 ug/day. The static test indicates that single point positioning precision of the GNSS receiver is better than 3 m. The sampling rate of the SINS is 200 Hz and the sample rate of GNSS receiver is 2 Hz. In this ship experiment, the GNSS data is post-processed with the Waypoint^®^ software (Version 8.6, produced by Novatel^®^, Calgary, AB, Canada and this software is bought by author’s group) to obtain position result. The process report from the software indicates that the precision of position results is better than 10 m in this ship experiment, which is much less than the position error of INS, then the position result from GNSS could be regarded as the position reference.

**Figure 14:**
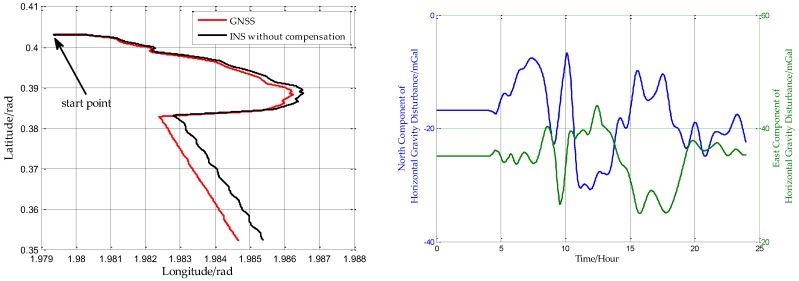
The (**left**) is the ship’s trajectory and the (**right**) is the horizontal gravity disturbance on the ship’s trajectory.

In this experiment, the ship was first at moor, then started to sail for about 24 h, as shown in Figure 14. The horizontal gravity disturbance on the ship’s trajectory is calculated based on EGM2008, and the position information used to calculate the horizontal gravity disturbance is from GNSS, and Figure 14 shows the horizontal gravity disturbance on the ship’s trajectory. The ship is first at moor in the first five hours, so the horizontal gravity disturbances are constants. After that, the ship started to sail, and the horizontal gravity disturbances correspondingly changed.

**Figure 15:**
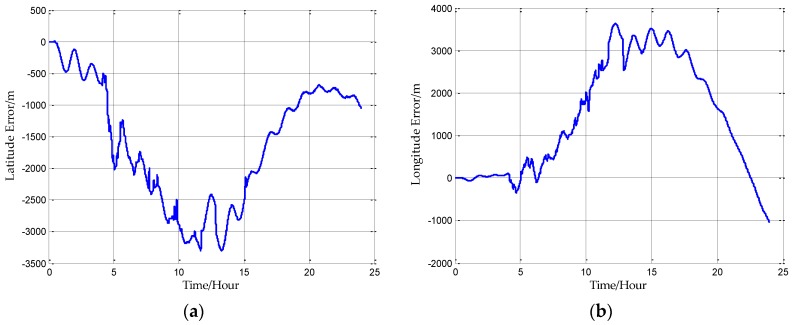
(**a**) is the latitude error without compensation and (**b**) is the longitude error without compensation.

Firstly, the INS data is processed without compensation, and the positioning error can be obtained with the GNSS reference. Figure 15a is the latitude error and Figure 15b is the longitude error. The main error characteristics of INS can be seen from Figure 15. The short period oscillation in the latitude error and longitude error is the Schuler oscillation, whose period is approximately 84.4 min. And the long period oscillation is the 24 h oscillation caused by the rotation of the Earth whose period is approximately 24 h. There is one more inconspicuous oscillation called Foucault oscillation, whose period is approximately 61.5 h at this latitude.

From Figure 15, the position errors of INS without compensation are less than 3 n miles in 24 h, and this is a high precision for INS. To further improve the precision of the INS, the proposed compensation methods are applied separately to the velocity calculation and attitude calculation. It should be noted that the compensations can only reduce the gravity-induced position error to some extent, and the position errors caused by initial error and inertial sensor biases can’t be eliminated by the compensations. In other words, the compensations can only reduce the error oscillations, but can’t completely eliminate these oscillations.

**Figure 16:**
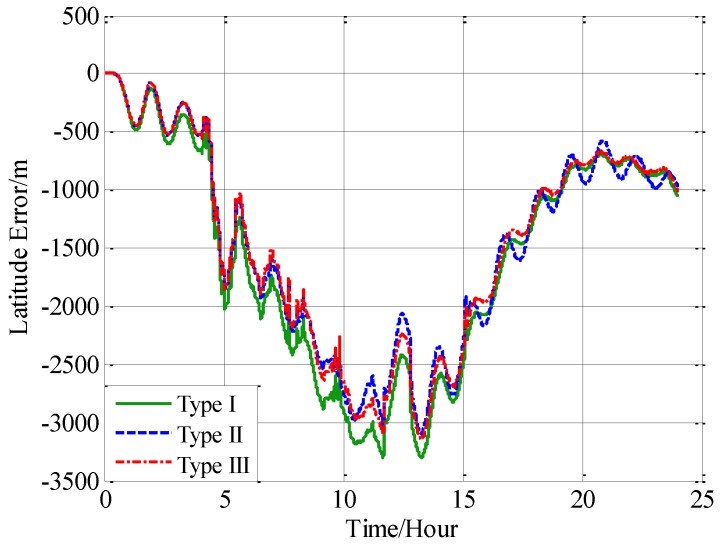
The comparison of the latitude error.

**Figure 17:**
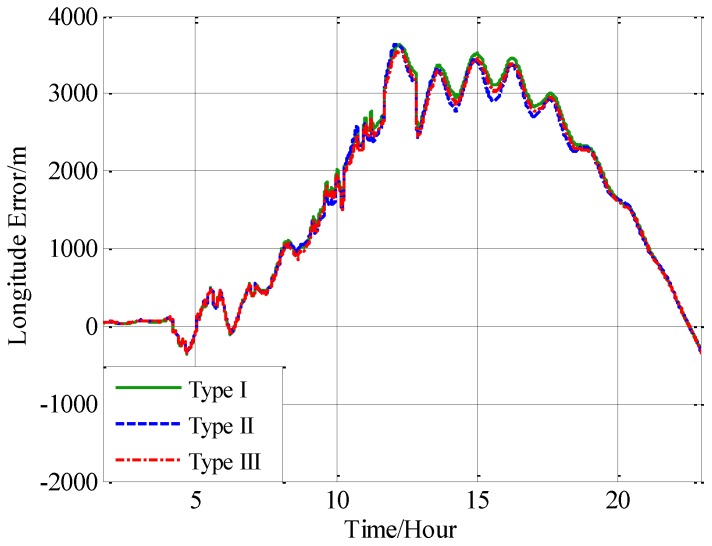
The comparison of the longitude error.

**Figure 18:**
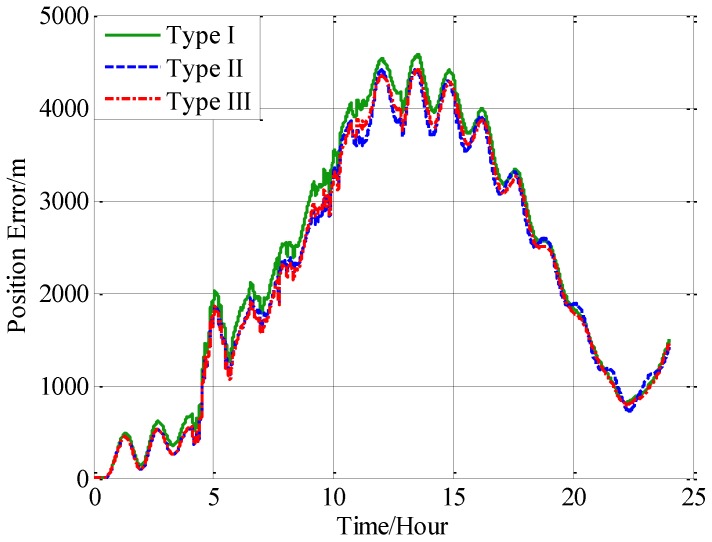
The comparison of the position error.

Three navigation results are compared here: the position errors obtained without compensation is denoted by type I, and the position errors obtained with compensation in velocity calculation is denoted by type II, and the position errors obtained with compensation in attitude calculation is denoted by type III. Figure 16 shows the latitude error comparison, and Figure 17 shows the longitude error comparison, and Figure 18 shows the position error comparison.

**Figure 19:**
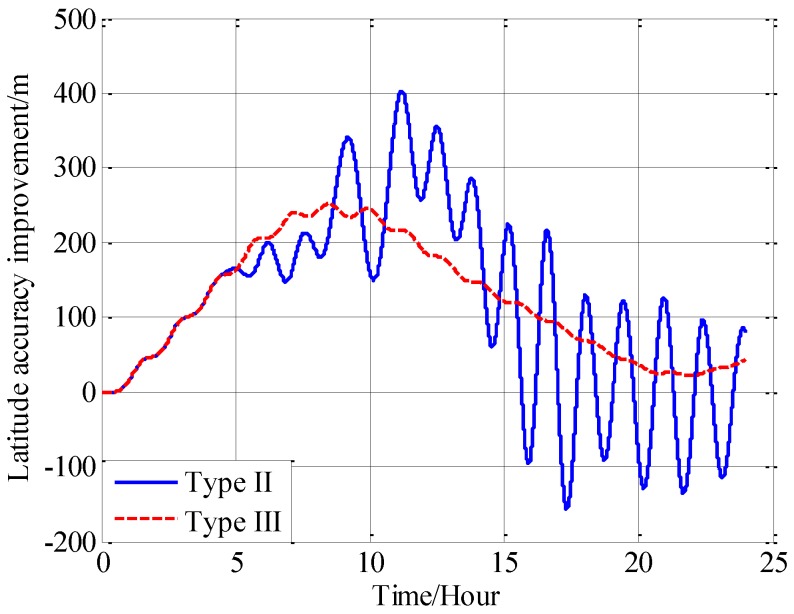
The precision improvement of latitude with compensation.

**Figure 20:**
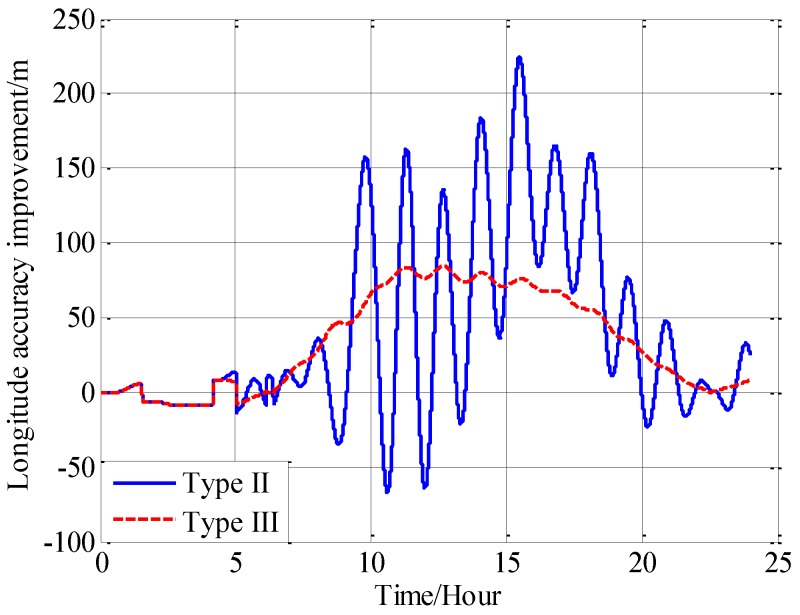
The precision improvement of longitude with compensation.

**Figure 21:**
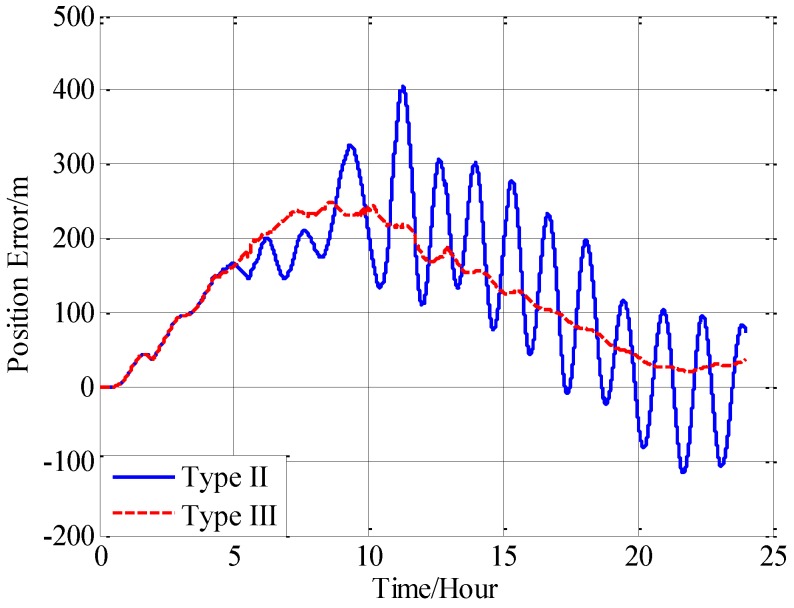
The precision improvement of position with compensation.

From Figure 16, Figure 17 and Figure 18, it can be found that both the proposed compensation methods can reduce the error oscillations of INS, which means the precision of the INS is improved after compensation. A quantitative evaluation of the accuracy improvement can be obtained by subtracting the positioning error of type II and III from type I, as shown in Figure 19, Figure 20 and Figure 21.

From the fifth hour to the twentieth hour, there is a great change in the horizontal gravity disturbance, and the values are also larger. That’s to say, horizontal gravity disturbance has a greater influence on INS in the fifth hour to the twentieth hour, then there is a greater potential for improvement in this period. That’s the reason why the positioning accuracy achieves a greater improvement from the fifth to the twentieth hour. During the last four hours, from the twentieth hour to the twentyfourth hour, the horizontal gravity disturbance changes slowly and becomes smaller, then the position improvement also decreases. And the maximum position accuracy improvement of compensation in velocity calculation is 405 m, about 10.07% increment, and the maximum position accuracy improvement of compensation in attitude calculation is 250 m, about a 9.77% increment.

Comparing the accuracy improvements of the two compensation methods, it can be found that the precision improvement of compensation in velocity calculation has a periodic oscillation whose period is approximately equal to Schuler period of the INS, the reason for this Schuler oscillation is that there exist some errors in the horizontal gravity disturbance calculated through the EGM2008. In fact, there also exists a Schuler oscillation in the precision improvement of compensation in the attitude calculation, but the oscillation amplitude is smaller, which is due to the different error characteristics of the velocity calculation and attitude calculation.

## 6. Conclusions

In this paper, from the perspective of coordinate system and vector calculation law, the effect of horizontal gravity disturbances on an INS is analyzed. Horizontal gravity disturbances will result in the navigation coordinate frame built in the initial alignment not being consistent with the navigation coordinate frame in which the navigation calculation is executed, and this mismatch of coordinate frame violates the vector calculation law and has an adverse effect on the accuracy of INS. Two compensation methods are proposed according to two navigation coordinate frame definitions, one of the methods implements the compensation in the velocity calculation, and the other does the compensation in the attitude calculation. The initial alignment simulation verifies that the navigation coordinate frames obtained in the initial alignment are consistent with the targets with compensations. A ship navigation experiment confirms the correctness and effectiveness of the proposed two methods, whereby a maximum positioning accuracy improvement of about 10% is achieved in the experiment.

## Figures and Tables

**Table 1:** Iterative algorithm of the fully normalized Legendre function.

Given: Latitude, Longitude and Height of the Calculated Point
*Step 1*: Substitute Equation (13) into Equation (7) to calculate ${\bar{P}}_{nn}$
*Step 2*: Substitute ${\bar{P}}_{nn}$ into Equation (8) to calculate ${\bar{P}}_{n,n-1}$
*Step 3*: Substitute ${\bar{P}}_{nn}$ and ${\bar{P}}_{n,n-1}$ into Equation (9) to calculate ${\bar{P}}_{nm}$
*Step 4*: Substitute ${\bar{P}}_{nn}$ into Equation (14) to calculate $d{\bar{P}}_{nn}\left(\cos\theta \right)/d\theta$
*Step 5*: Substitute ${\bar{P}}_{nm}$ into Equation (15) to calculate $d{\bar{P}}_{nm}\left(\cos\theta \right)/d\theta$

**Table 2:** Inertial sensor performance ranges.

Inertial Sensor	Performance Units	Performance Ranges		
		High	Medium	Low
Gyroscope	degree/h	$\leq 10^{-3}$	$\approx 10^{-2}$	$\geq 10^{-1}$
Accelerometer	$g\approx 9.8{\;m/s}^{2}$	$\leq 10^{-8}$	$\approx 10^{-6}$	$\geq 10^{-5}$

**Table 3:** Initial states of the INS.

	Units	Initial State	Initial Value
Initial attitude	degree	roll	5
		pitch	−3
		yaw	−115
Initial velocity	m/s	North velocity	0
		East Velocity	0
		Downward velocity	0
Initial position	degree	latitude	23
	degree	longitude	113
	m	height	9.5

**Table 4:** When n frame is the navigation coordinate frame, estimation accuracy comparison of the simulations, unit: degree.

Euler Angle	True Value	Without Compensation		Compensation in Velocity Calculation	
		Estimation Result	Estimation Error	Estimation Result	Estimation Error
Roll	5	4.99834	−0.00166	5.00002	0.00002
Pitch	−3	−2.99853	0.00147	−2.99999	0.00001
Yaw	−115	−115.03915	−0.03915	−115.01628	−0.01628

**Table 5:** When $n^{\prime }$ frame is the navigation coordinate frame, estimation accuracy comparison of the simulations, unit: degree.

Euler Angle	True Value	Without Compensation		Compensation in Attitude Calculation	
		Estimation Result	Estimation Error	Estimation Result	Estimation Error
Roll	5	4.99979	−0.00021	4.99981	−0.00019
Pitch	−3	−3.00009	−0.00009	−3.00002	−0.00002
Yaw	−115	−114.95142	0.04858	−114.97197	0.02803
